# Accelerating 3D Convolutional Neural Network with Channel Bottleneck Module for EEG-Based Emotion Recognition

**DOI:** 10.3390/s22186813

**Published:** 2022-09-08

**Authors:** Sungkyu Kim, Tae-Seong Kim, Won Hee Lee

**Affiliations:** 1 Department of Software Convergence, Kyung Hee University, Yongin 17104, Korea; 2 Department of Biomedical Engineering, Kyung Hee University, Yongin 17104, Korea

**Keywords:** emotion recognition, affective computing, convolutional neural network, EEG, DEAP, deep learning

## Abstract

Deep learning-based emotion recognition using EEG has received increasing attention in recent years. The existing studies on emotion recognition show great variability in their employed methods including the choice of deep learning approaches and the type of input features. Although deep learning models for EEG-based emotion recognition can deliver superior accuracy, it comes at the cost of high computational complexity. Here, we propose a novel 3D convolutional neural network with a channel bottleneck module (CNN-BN) model for EEG-based emotion recognition, with the aim of accelerating the CNN computation without a significant loss in classification accuracy. To this end, we constructed a 3D spatiotemporal representation of EEG signals as the input of our proposed model. Our CNN-BN model extracts spatiotemporal EEG features, which effectively utilize the spatial and temporal information in EEG. We evaluated the performance of the CNN-BN model in the valence and arousal classification tasks. Our proposed CNN-BN model achieved an average accuracy of 99.1% and 99.5% for valence and arousal, respectively, on the DEAP dataset, while significantly reducing the number of parameters by 93.08% and FLOPs by 94.94%. The CNN-BN model with fewer parameters based on 3D EEG spatiotemporal representation outperforms the state-of-the-art models. Our proposed CNN-BN model with a better parameter efficiency has excellent potential for accelerating CNN-based emotion recognition without losing classification performance.

## 1. Introduction

Emotion is a mental and physiological state that results in physical and psychological changes that affect thought and behavior [1]. Emotions are often thought to be consciously experienced and intentional [2]. Artificial emotional intelligence, also known as affective computing, is an emerging technology that allows computers and systems to identify, process, and understand human emotions [3]. Emotion recognition has received significant attention in multiple areas, such as human–computer interaction (HCI) [4], cognitive neuroscience [5], disease detection [6], virtual reality [7], and robotics [8]. Emotion recognition is defined as identifying human emotions and is often conducted using non-physiological data such as facial expressions [6,9], voice [10], and body movement [11]. On the other hand, recent studies have focused on the analysis of physiological signals including galvanic skin resistance (GSR) [12], electrocardiography (ECG) [13], electromyography (EMG) [14], and electroencephalography (EEG) [15,16,17,18,19,20,21,22,23,24,25,26,27]. Compared to non-physiological data, these data are capable of reflecting a real emotional state objectively, providing a reliable way to identify real emotions [28,29].

Among the physiological modalities, EEG signals have become increasingly attractive for detecting human emotions due to their relative simplicity for collecting data and their objective evaluation of emotions [28,29]. Moreover, EEG signals encompass various spatial, temporal, and spectral information about different emotions evoked by specific stimulation paradigms [30]. Traditional emotion recognition techniques based on machine learning typically extract handcrafted features from EEG signals, and then these features are entered into machine learning classifiers such as a support vector machine, random forests, and k-nearest neighbors for emotion recognition [31]. EEG features can be extracted from the time, frequency, and time-frequency domains (see review, [32]). However, classical machine learning models require the process of extracting emotional features and selecting the most relevant features, limiting their performance for EEG-based emotion recognition. 

Recently, a great variety of deep learning approaches have been proposed to improve the performance of emotion recognition using EEG signals. The deep learning models, most commonly based on convolutional neural networks (CNN) and long short-term memory (LSTM) networks, automatically learn deep features and recognize emotions [15,16,19,20,21,22,23,24,25,26,27]. With the advent of various deep learning approaches, researchers have attempted to improve the accuracy of emotion recognition using different EEG features. For example, previous studies have shown that temporal information captured by conventional an LSTM network is useful for enhancing emotion recognition using EEG [33]. Recent studies have used traditional CNN in order to capture spatial information among different EEG channels [34]. The spectral features such as the power spectral density (PSD) and differential entropy (DE) in the EEG signals were also used for emotion recognition [35]. Despite a wide range of possible EEG features, most studies have only focused on one particular type of EEG feature or a combination of two features such as spatial-temporal and spatial-spectral information for emotion recognition [15,16,17,18,19,20,21,22,23,24,25,26]. The exiting models did not make full use of different EEG features, potentially limiting the performance of the deep learning models using EEG. In addition, various deep learning models based on different EEG feature types have delivered superior accuracies in EEG-based emotion recognition, but it comes at the cost of a high computational complexity. 

In this study, we propose a novel three-dimensional (3D) CNN model integrated with the channel bottleneck module, named CNN-BN, for EEG-based emotion recognition. We applied bottleneck building blocks to the CNN architecture with the goal of reducing computational costs while preserving classification accuracy. We evaluated the performance of our proposed model for emotion recognition using the publicly available EEG dataset from the database for emotional analysis using physiological signals (DEAP) [36]. We generated a 3D spatiotemporal representation of EEG signals as the input of our model. The CNN-BN model extracts spatial-temporal EEG features based on the constructed 3D spatiotemporal representation of EEG signals and predicts different emotional states. We assessed the classification accuracy of the proposed model in the valence and arousal classification tasks. We further compared the CNN-BN model to the LSTM model and regular CNN model considered as baseline models, which are widely used in approaches concerning EEG-based emotion recognition [28,37,38]. The main contributions of this paper are summarized as follows:

- We propose a novel 3D CNN model integrated with the channel bottleneck module (CNN-BN) based on the constructed 3D EEG representation.
- Extensive experiments are conducted on the DEAP dataset for the valence and arousal classification tasks. The experimental results show that our CNN-BN model outperforms baseline and state-of-the-art-models and significantly reduces computational complexity.
- Our CNN-BN model with a better parameter efficiency has an excellent potential for accelerating CNN-based emotion recognition without losing classification performance.

## 2. Related Work

In recent years, deep learning approaches have been widely used for EEG-based emotion recognition due to their ability to learn data representation by using multiple hidden layers in the neural network as opposed to traditional machine learning [39]. Deep learning models are trained by using large, labeled datasets and neural network architectures that automate feature learning without the need for manual feature extraction. 

Typically, studies using deep learning techniques extract temporal features, spectral features, spatial features, or a combination of different features from EEG signals for emotion recognition. For example, for temporal feature extraction, Alhagry et al. [15] proposed an LSTM model to recognize emotion from raw EEG signals. They used raw EEG signal segments with a length of 5 s. The LSTM model contains two LSTM layers and one dense layer. They achieved an average accuracy of 85.45%, 85.65%, and 87.99% in the binary classification of valence, arousal, and liking, respectively.

For spectral feature extraction, Wang et al. [16] proposed a residual block-based deep CNN with electrode-frequency distribution maps (EFDM). They achieved an average accuracy of 90.59% in the three labeled emotional states (negative, neutral, and positive) on the SEED dataset. They also applied this pre-trained model to the DEAP dataset for testing the performance of emotion classification, resulting in an average accuracy of 82% for the three labeled emotion states’ (negative, neutral, and positive) classification. 

For spectral-temporal feature extraction, Yin et al. [17] proposed a multiple fusion layer-based ensemble classifier of a stacked autoencoder (MESAE) for emotion recognition. They developed a model structural identification index to find a parsimonious emotion classifier. To this end, they extracted several features such as power features, power difference, and temporal features from the 6-second length of each EEG segment. They achieved an average accuracy of 76.17% and 77.19% in the binary classification of valence and arousal, respectively, on the DEAP dataset. Fang et al. [18] proposed a multi-feature deep forest (MFDF) model that divides the EEG signal into several EEG frequency bands and then extracts the PSD and DE from each frequency band and the original signal as features. They achieved an average accuracy of 71.05% in the five-class emotion (neutral, angry, sad, happy, and pleasant) classification. Sharma et al. [19] proposed an LSTM model using third-order cumulants (ToC). Before applying the ToC, a discrete wavelet transform (DWT) is used to decompose the EEG signal into five frequency sub-bands and a particle swarm optimization algorithm is used to optimize the feature matrix. They achieved an average accuracy of 84.16%, 85.21%, and 82.01% in the binary classification of valence, arousal, and four labeled emotion classes (high valence and high arousal, high valence and low arousal, low valence and high arousal, and low valence and low arousal), respectively, on the DEAP dataset. An et al. [20] proposed 3D feature fusion and convolutional autoencoder (CAE). The 3D feature fusion fuses the DE features of different frequency bands of EEG signals to construct the 3D features of EEG signals that contain the spatial information between channels. Their recognition accuracies for valence and arousal was 89.49% and 90.76% on the DEAP dataset, respectively. Islam et al. [21] constructed EEG-based functional connectivity maps using the Pearson’s correlation coefficient between the EEG signals of multichannel EEG frequency sub-bands. These feature maps were converted into images and fed into the CNN model as inputs to recognize the emotional states. The maximum accuracy of 78.22% for valence and 74.92% for arousal were achieved on the DEAP dataset.

Regarding spatiotemporal feature extraction, Liu et al. [22] proposed an effective multi-level feature guided capsule network (MLF-CapsNet). As an end-to-end framework, MLF-CapsNet can simultaneously extract features from the raw EEG signals and determine their emotional states. They achieved an average accuracy of 97.97%, 98.31%, and 98.32% for valence, arousal, and dominance, respectively, on the DEAP dataset, and achieved 94.59%, 95.26%, and 95.13% for valence, arousal, and dominance, respectively, on the DREAMER dataset. Sartipi et al. [23] proposed a spatial-temporal attention neural network (STANN), a parallel structure of the multi-column CNN, and an attention-based bidirectional LSTM to extract the discriminative spatial and temporal features of EEG signals. Additionally, the inter-channel relationships of EEG signals were explored using graph signal processing (GSP) tools. They achieved an average accuracy of 94.8%, 96.1%, and 92.7% in the binary classification of valence, arousal, and the four labeled emotional states (high valence and high arousal, high valence and low arousal, low valence and high arousal, and low valence and low arousal), respectively, on the DEAP dataset. Yin et al. [24] proposed a fusion model of graph convolutional neural networks (GCNN) and LSTM. For this model, the EEG signal is segmented into a 6 s time window, and then differential entropy is extracted from each segment to construct a feature cube. The feature cube of each segment serves as the input of the GCNN and LSTM. They achieved an average accuracy of 84.81% and 85.27% in the binary classification of valence and arousal, respectively, on the DEAP dataset. Ding et al. [25] proposed the TSception method, which consists of three layers: the dynamic temporal layer, which finds temporal information from the input data by using multi-scale 1D convolution; the asymmetric spatial layer, which finds spatial information by using the multi-scale 1D convolution kernel; the fusion layer, which fuses these spatial and temporal data with 1D convolution. They achieved an average accuracy of 59.14% and 61.57% in the binary classification of valence and arousal, respectively, on the DEAP dataset, and 61.27% and 60.61% for valence and arousal on the MAHNOB-HCI dataset. 

For spatio-spectral feature extraction, Chao et al. [26] proposed a framework that contains a multiband feature matrix (MFM) and a capsule network (CapsNet) for classification. The MFM extracts band-wise spatial PSD and the CapsNet finds spatial-spectral information. They achieved an average accuracy of 66.73%, 68.28%, and 67.25% in the binary classification of valence, arousal, and dominance, respectively, on the DEAP dataset.

For spatial-spectral-temporal feature extraction, Jia et al. [27] proposed the HetEmotionNet, which is a two-stream heterogeneous graph recurrent neural network. HetEmotionNet includes a spatial-temporal graph representation and a spatial-spectral graph representation. They achieved an average accuracy of 97.66% and 97.30% in the binary classification of valence and arousal, respectively, on the DEAP dataset, and 93.95% and 93.90% on the MAHNOB-HCI dataset.

## 3. Methodology

### 3.1. Model Overview

**Figure 1:**
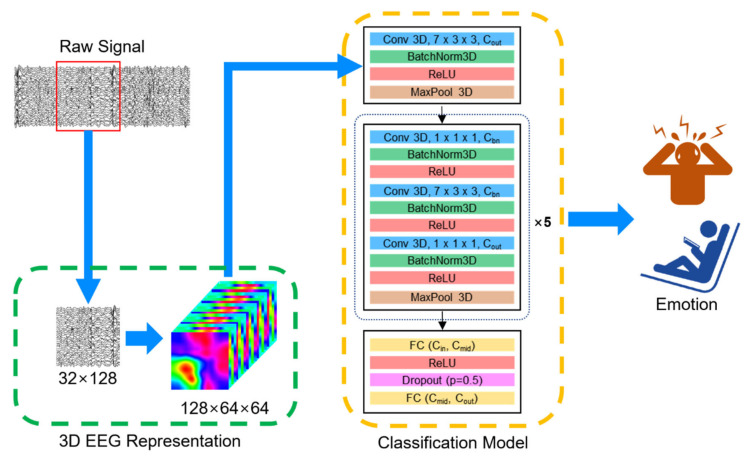
Overview of the workflow for EEG-based emotion recognition. 3D representation of EEG signals is constructed as the input of our proposed CNN-BN model. The model consists of a convolutional block, five consecutive bottleneck blocks, and a dense block for EEG-based emotion recognition. Conv, BatchNorm, MaxPool, and FC represent the convolution, batch normalization, max pooling, and fully connected layer, respectively. $C_{\mathrm{in}}$ and $C_{\mathrm{out}}$ are the input and output channels at each layer, respectively. $C_{\mathrm{bn}}$ denotes the reduced channel within a bottleneck block. $C_{\mathrm{mid}}$ is the reduced channel by the first fully connected layer. ReLU represents the rectified linear unit activation function.

Figure 1 illustrates the overall structure of our model. We proposed a novel 3D CNN model integrated with the channel bottleneck module (CNN-BN) based on 3D spatiotemporal representation of EEG signals. We constructed 3D spatiotemporal representation of EEG signals, providing the spatial distribution of temporal information of EEG signals. Our proposed CNN-BN model consists of a convolution block, five consecutive bottleneck blocks, and a dense block. The bottleneck block was introduced into the CNN architecture for the purpose of improving the computational and memory-related efficiency of the 3D convolutions. 

### 3.2. 3D Representation

**Figure 2:**
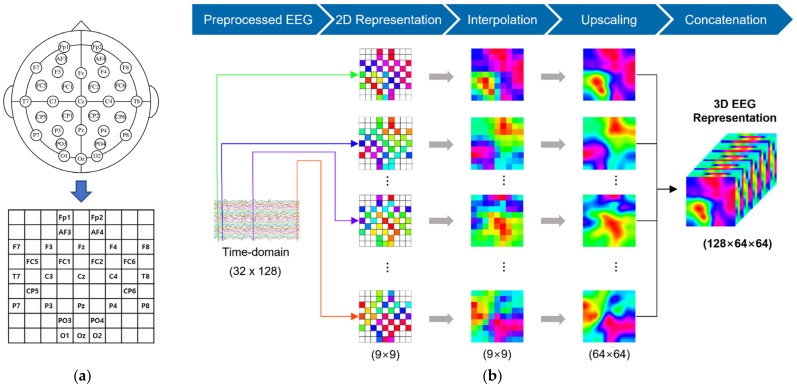
(**a**) 2D representation of 32 channels according to the international 10–20 system. The names of the electrodes are annotated in the 2D map. The EEG signals at a time point from all EEG channels are transformed into a 2D map (9 × 9 matrix) according to the positions of electrodes on the brain. (**b**) The process of 3D map representation of EEG signals. The 3D EEG spatiotemporal representation serves as the input of the proposed CNN-BN model.

In the experiment, we generated 3D spatiotemporal representation of EEG signals as the input of the proposed model. To construct the 3D spatiotemporal representation, the EEG signals at a time point (1-s non-overlapping window) from all EEG channels were transformed into a 2D map (9 × 9 matrix) according to the positions of electrodes on the brain (Figure 2a). This preserves the topology of different electrodes. Then, radial basis function (RBF) interpolation [40] was applied to fill in null values where the corresponding electrodes were not available. To improve the resolution of the 2D EEG feature maps, bicubic interpolation was employed to create the EEG feature maps with dimensions of 64 × 64. Bicubic interpolation was chosen as it allows for smoother resampling with fewer image artifacts compared to nearest-neighbor or bilinear interpolation [41]. Finally, a series of 2D maps from all time points was stacked to form the 3D spatiotemporal representation. The process for generating the 3D EEG representation is shown in Figure 2b.

### 3.3. Convolutional Neural Network with Channel Bottleneck Module

Our proposed CNN-BN model was set up to take the 3D EEG representation data (128 × 64 × 64) to learn the spatiotemporal EEG features as the input. The proposed CNN-BN architecture consists of a convolutional block, five consecutive bottleneck blocks, and a dense block. We designed our CNN-BN to have 16 convolution layers, 16 batch normalization layers, 17 rectified linear unit (ReLU) activation functions, 6 max pooling layers, and by 2 fully connected layers. The network architecture is presented in Figure 1 and detailed in Table 1. We will describe the details of each part in sequence.

#### 3.3.1. Convolution Block

A convolution block consists of a 3D convolution layer; a 3D batch normalization, followed by the ReLU activation function; and a max pooling layer. In a convolution layer, the 3D convolution kernel is set to 7 × 3 × 3 with stride 1 for the spatiotemporal convolutions. Note that 3D convolution and pooling kernels have a size of *d* × *k* × *k*, where *d* is the kernel’s temporal length and *k* is the kernel’s spatial size. The pooling operation was applied with a kernel size of 2 × 1 × 1, which reduces the output dimension from the convolution layer to decrease the computational complexity and prevent overfitting. We used the max pooling operation with a stride of 2 × 1 × 1 that selects only the maximum value in each feature map and consequently reduces the temporal dimension of the output data by a factor of 2, compared to the input data. 

#### 3.3.2. Bottleneck Module

**Figure 3:**
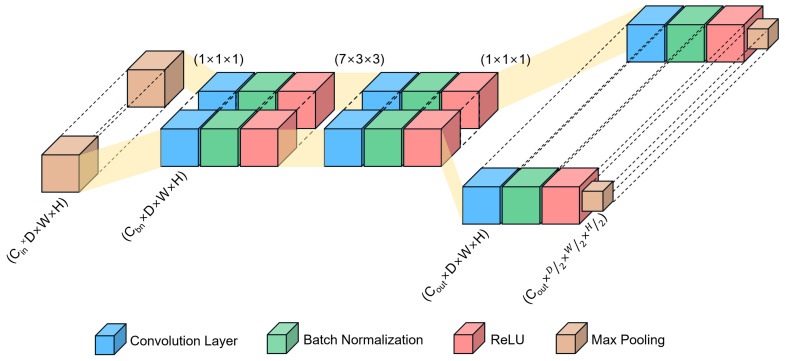
Illustration of a channel bottleneck block. $C_{\mathrm{in}}$, $C_{\mathrm{bn}}$, and $C_{\mathrm{out}}$ represent the number of input channels, reduced channels, and output channels within the channel bottleneck block, respectively. D, W, and H denote the length, width, and height of the feature map, respectively. The first 1 × 1 × 1 convolution reduces the number of channels in the input data from the preceding layer. The 7 × 3 × 3 convolution is then computed on the reduced-channel layer. Finally, the last 1 × 1 × 1 convolution increases the number of channels in the output data.

A schematic diagram for a channel bottleneck block is presented in Figure 3 and detailed in Table 1. A bottleneck module consists of five consecutive bottleneck blocks. For each bottleneck block, we used a stack of three convolutional layers. These three layers are 1 × 1 × 1, 7 × 3 × 3, and 1 × 1 × 1 convolutions. In our setting, the 1 × 1 × 1 convolutions have a dual purpose: 1 × 1 × 1 layers are responsible for reducing and then increasing (restoring) dimension, leaving 7 × 3 × 3 a bottleneck with smaller input or output dimensions. In the channel bottleneck block, a 1 × 1 × 1 convolution is first applied to reduce the number of channels to 1/4 and then a regular 7 × 3 × 3 convolution is computed on the reduced channel layer, followed by another 1 × 1 × 1 convolution to restore the desired number of channels (e.g., the original size). Specifically, the first convolutional layer in the bottleneck blocks reduces the number of channels by a factor of 4. The third convolutional layer in the first and second bottleneck blocks increases the number of channels by a factor of 8, while they are increased by a factor of 4 in the third to fifth bottleneck blocks. The number of channels for the first and second convolutional layers in the bottleneck blocks from 1 to 5 are 16, 32, 64, 64, and 64, respectively. The number of channels for the third convolutional layer from 1 to 5 are 128, 256, 256, 256, and 256, respectively. For example, as shown in Figure 3, in the first bottleneck block, the first convolutional layer reduces the number of channels to 16 from 64. The second convolutional layer extracts the spatiotemporal features while maintaining 16 channels. Subsequently, the third convolutional layer increases the number of channels to 128. This bottleneck structure allows for decreasing not only the number of parameters required for the convolutions but also the computational costs without significant performance penalty. Each convolutional layer is followed by a 3D batch normalization and a ReLU activation function. All pooling layers employ max pooling with a kernel size of 2 × 2 × 2 with a stride of 2 × 2 × 2, which means the size of output data is reduced by a factor of 2 compared with the input data from the preceding layers. 

#### 3.3.3. Dense Block 

A dense block is implemented with two fully connected layers with a ReLU activation function and a dropout layer. Two fully connected layers are used for emotion classification based on the features extracted by previous layers. The output of the last bottleneck block is flattened and fed into the fully connected layers. Here, we used a dropout of 0.5 to reduce the network complexity. The last-stage fully connected layer predicts the probabilities of each emotion label. 

## 4. Experiments

### 4.1. Dataset

**Figure 4:**
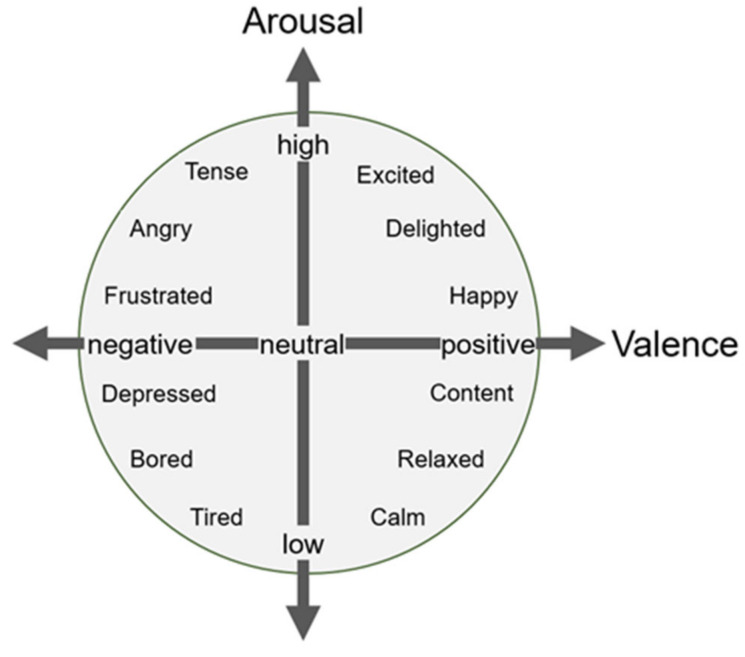
Valence–arousal model for emotional representation.

The DEAP is a multimodal dataset that includes EEG and peripheral physiological signals of 32 participants (age range = 19–37 years; mean age = 26.9 years; 16 females) who watched 40 excerpts of one-minute duration music videos. The EEG signals were recorded at a sampling rate of 512 Hz from 32 active electrodes (channels) according to the international 10–20 system (Figure 2a), while the peripheral physiological signals (8 channels) include the galvanic skin response, skin temperature, blood volume pressure, respiration rate, electromyogram, and electrooculogram (horizontal and vertical). Each participant rated their levels of arousal (passive/active), valence (negative/positive), liking (like/dislike), and dominance using self-assessment manikins (SAM). Participants selected the numbers 1–9 for their emotional state for each trial. The 2D emotion space is depicted in Figure 4. The valence measures the pleasantness of an emotion and arousal measures the intensity of an emotion [42]. The EEG signals were down-sampled to 128 Hz, electrooculogram (EOG) artifacts were removed, and a bandpass filter between 4 and 45 Hz was applied. We extracted the last 60 s stimulus-related signals and removed the first 3 s stimulus-independent signals for each trial. More details on the DEAP dataset are provided in [36]. 

### 4.2. Baseline Models

We compared the classification performance of our proposed CNN-BN model to the LSTM [37] and regular 3D CNN model (C3D [38]), which are the most commonly used methods for emotion recognition. We chose to use the LSTM to examine the effectiveness of extracting the spatiotemporal EEG features and the regular 3D CNN model (C3D) to investigate the impact of integrating the channel bottleneck module into the 3D CNN architecture on the classification performance. For a fair comparison to the baseline models, we performed the same data processing and experimental settings for all methods. 

#### 4.2.1. Regular Convolutional Neural Network

To test for the effectiveness of channel bottleneck blocks in CNN, we adopted a regular 3D CNN model without a channel bottleneck block. Our plain baseline was mainly inspired by a popular 3D CNN approach, namely, convolutional 3D (C3D) [38], which consists of eight convolution layers, five max pooling layers, two fully connected layers, and a softmax output layer. This C3D network was originally developed for learning the spatiotemporal features from videos, where the 3D convolution kernel was set to 3 × 3 × 3 at all convolution layers. 

**Figure 5:**
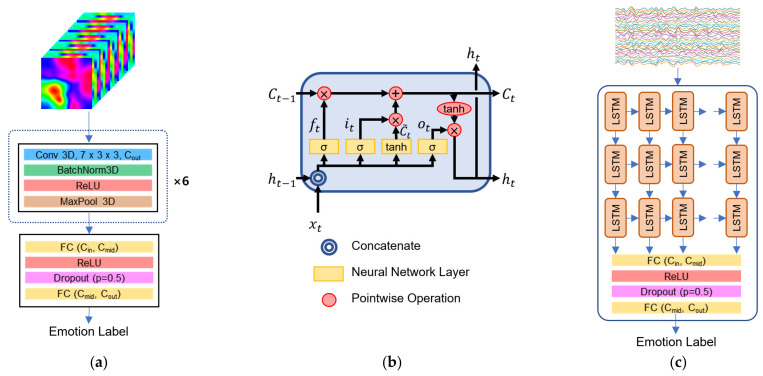
(**a**) 3D CNN architecture without a channel bottleneck; (**b**) Long Short-Term Memory (LSTM) cell architecture; (**c**) LSTM model architecture. Conv, BatchNorm, MaxPool, and FC represent the convolution, batch normalization, max pooling, and fully connected layer, respectively. $C_{\mathrm{in}}$ and $C_{\mathrm{out}}$ are the input and output channels at each layer, respectively. $C_{\mathrm{mid}}$ is the reduced channel by the first fully connected layer. ReLU represents the rectified linear unit activation function.

To apply the C3D approach to the 3D EEG representation data (128 × 64 × 64) for emotion recognition, we modified the kernel size as 7 × 3 × 3 at a convolution layer due to the temporal dimension being higher than the spatial dimension of the 3D EEG data. Figure 5a shows a plain, regular 3D CNN architecture without a channel bottleneck block. The model consists of six consecutive 3D convolution blocks and two fully connected layers. 

#### 4.2.2. Long Short-Term Memory (LSTM)

We compared the CNN-BN model to the LSTM network, which is a sequential network [37]. LSTM network is a special kind of recurrent neural network (RNN) designed to model temporal sequences and their long-range dependencies. It is capable of handling the vanishing gradient problem faced by RNN [37]. The RNN cell is replaced by the LSTM cell, which can remove or add information to the cell state, carefully regulated by structures called gates. The LSTM cell consists of a forget gate, an input gate, and an output gate (Figure 5b). In a cell of the LSTM network, the first gate is a forget gate to decide whether to keep the information from the previous timestamp or forget it. The second gate is an input gate used to quantify the importance of the new information carried by the input. Finally, the output will be based on the updated cell state and a sigmoid layer that decides which parts of the cell state will be the final output. 

In this paper, we designed the LSTM model, which consists of three LSTM layers and two fully connected layers, to predict different emotion labels (Figure 5c). The EEG signals were segmented using a 1-second non-overlapping window as in the CNN-BN. Every LSTM layer is followed by a hyperbolic tangent (tanh) activation function. The output map after three LSTM layers is flattened and fed into the first fully connected layer with a ReLU activation function. The dropout layer with a value of 0.5 is applied to prevent overfitting. The last-stage fully connected layer predicts the different emotion class labels. 

### 4.3. Experimental Settings

Our model was implemented with the PyTorch framework and trained on a NVIDIA RTX A6000 GPU. The cross-entropy was used as loss function [43], which is defined as follows:

(1) $$L=-\frac{1}{N}\overset{N}{\underset{i=1}{\sum }}(y_{i}log\left(\hat{y_{i}}\right)+\left(1-y_{i}\right)log\left(1-\hat{y_{i}}\right))$$

where *N* is the number of samples, $y_{i}$ is the classification of the *i*-th sample (0 or 1), and $\hat{y_{i}}$ is the predicted probability of the *i*-th sample being recognized. A stochastic gradient descent (SGD) optimizer was applied. We trained the networks using a mini-batch size of 32 with an initial learning rate of 0.01. The learning rate was decayed by a factor of 0.9 after every 10 epochs. The training was stopped after about 90 epochs. 

We divided the continuous arousal and valence dimensions into two categorical levels (low/high) with the threshold of five. The DEAP dataset contains continuous levels of arousal, valence, liking, dominance, and familiarity. Here, we focused on the arousal and valence dimensions in this study, which are the most common components in emotion models (Figure 4) [15,17,19,20,21,22,23,24,25,26,27]. We performed two binary (arousal-level and valence-level) classification tasks using a cross-validation strategy. The 3D spatiotemporal representation data were randomly split into a training set (80%) for model training and a testing set (20%) for model evaluation. We repeated this procedure five times to validate the classification performance of the models. 

### 4.4. Performance Evaluation Metrics

To evaluate the classification performance of the model, we used the following evaluation metrics: recall, precision, F1-score, and accuracy, defined as:

(2) $$\mathrm{Recall}=\frac{\mathrm{TP}}{\mathrm{TP}+\mathrm{FN}}$$

(3) $$\mathrm{Precision}=\frac{\mathrm{TP}}{\mathrm{TP}+\mathrm{FP}}$$

(4) $$F1-\mathrm{score}=2\times \frac{\mathrm{Precision}\times \mathrm{Recall}}{\mathrm{Precision}+\mathrm{Recall}}$$

(5) $$\mathrm{Accuracy}=\frac{\mathrm{TP}+\mathrm{TN}}{\mathrm{TP}+\mathrm{FP}+\mathrm{TN}+\mathrm{FN}}$$

where TP, TN, FN, and FP denote true positives, true negatives, false negatives, and false positives, respectively.

## 5. Results and Discussion

The present study proposed the CNN-BN model for EEG-based emotion recognition using 3D EEG spatiotemporal representation data. We developed a model that uses less memory and is computationally more efficient. We conducted comprehensive evaluations of the proposed model by comparing its performance with baselines and state-of-the-art models reported in prior studies. The performance of our CNN-BN model is superior to the baseline models tested and the state-of-the-art performance with respect to classifying different emotion class labels. 

### 5.1. Classification Performance

**Figure 6:**
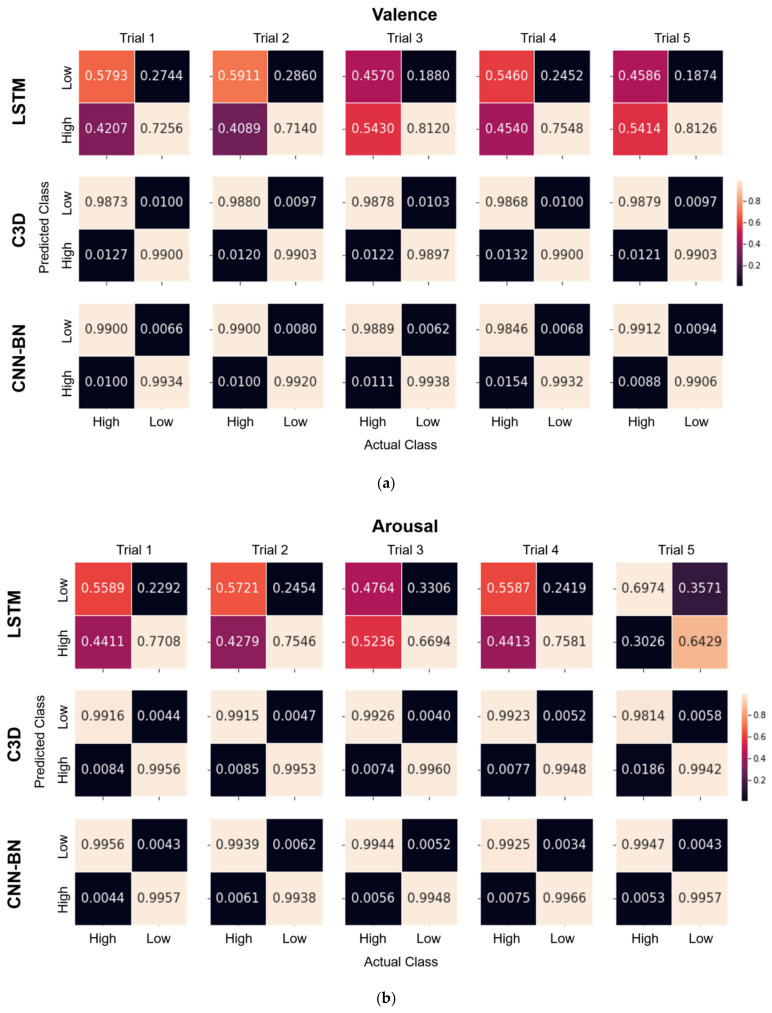
Confusion matrices of the binary emotion classification for (**a**) valence and (**b**) arousal for every trial (5 trials) for the LSTM model (top) and C3D model (middle) considered as baselines and the proposed CNN-BN model (bottom).

We compared our proposed CNN-BN model with two baseline models on the DEAP dataset. In Figure 6, the confusion matrices of binary emotion classification (valence-level and arousal-level) are separately displayed for each of the five trials for the LSTM, C3D, and CNN-BN models. The results indicate that the proposed CNN-BN model performed best over all the experimental trials compared to the other baselines. Table 2 presents the average accuracy and standard deviation of these models for the valence and arousal classification. The experimental results show that our model achieved the best performance on the DEAP dataset. The LSTM model performed markedly worse compared to the other models, which had an average accuracy of 66.51% and 65.08% (recall = 66.00% and 64.59%; precision = 66.41% and 64.91%; F1-score = 65.79% and 64.25%) in the binary classification of valence and arousal, respectively. The C3D model had an average accuracy of 98.90% and 99.29% (recall = 98.88% and 99.25%; precision = 98.88% and 99.30%; F1-score = 98.88% and 99.28%) for valence and arousal, respectively, but a slightly lower performance compared to the proposed CNN-BN model. Our model performed best with the highest accuracy of 99.10% and 99.48% (recall = 99.08% and 99.48%; precision = 99.10% and 99.47%; F1-score = 99.09% and 99.47%) for valence and arousal, respectively. Our results indicate that the CNN-BN model achieved a superior performance compared to the LSTM and C3D models, specifically, an improvement of 32.59% and 0.20% for valence and of 34.40% and 0.19% for arousal, respectively. This study suggests that the CNN-BN approach adequately extracts the spatiotemporal features in multi-channel EEG signals, which enables an accurate differentiation between different emotional states. 

### 5.2. Effects of Channel Bottleneck Blocks in CNN

We examined the effects of integrating channel bottleneck blocks into the CNN by comparing the number of parameters and the number of floating-point operations (FLOPs) between the C3D and CNN-BN models. Table 3 presents the number of parameters and the FLOPs in the C3D and CNN-BN models. The number of parameters were 16.05 M and 1.11 M for the C3D and CNN-BN models, respectively. These results demonstrate the effectiveness of channel bottleneck blocks with respect to the number of parameters. In our network, the bottleneck blocks reduced the number of channels by a quarter using a cheap 1 × 1 × 1 convolution, so that the following 7×3×3 convolution had fewer parameters. In contrast, the 7 × 3 × 3 convolution in the C3D was the most computationally expensive module, limiting the acceleration of the CNN’s computation. Our results show that channel bottleneck blocks reduced the number of parameters by 93.08%. Moreover, the FLOPs of the C3D and CNN-BN models were 449.29 G and 22.74 G, respectively. Channel bottleneck blocks reduced the FLOPs by 94.94% compared to the C3D. Our CNN-BN model also yielded a faster inference time (7.44 ms) than the C3D model (15.77 ms). Taken together, these results suggest that the channel bottleneck blocks are capable of reducing the model size and time complexity. The CNN-BN model with fewer parameters has an excellent potential for accelerating CNN-based emotion recognition without a significant loss in classification performance. 

We showed that the simple bottleneck module proposed in this study, inspired by previous studies [44,45,46], can efficiently reduce computational complexity and lead to better classification performance. A bottleneck block has been often used in several neural network architectures such as GoogLeNet [44], ResNet [45], and DenseNet [46] in order to encourage the networks to reduce the number of feature maps in the network, which otherwise tend to increase in each layer. In the present study, this was achieved by using 1 × 1 × 1 convolutions with fewer output channels than input channels. The bottleneck blocks help reduce the number of parameters in the network and the computation time while still allowing it to have depth and represent many feature maps [44,45,46]. Our work is related to other CNN architectures (e.g., FCN [47], UNet [48], SegNet [49], and ENet [50]) based on an encoder–decoder architecture [51]. The encoder architecture is identical to vanilla CNN, which is composed of several convolution layers followed by max-pooling layers. The encoder layers perform feature extraction of the down-sampled object. On the other hand, the decoder layers perform up-sampling after each convolutional layer to compensate the down-sampling effects of the encoder, and to generate an output with the same size as the input. The decoder network can have a small number of layers for reducing the computational load. 

### 5.3. Performance Comparison with the State-of-the-Art Models

We compared the performance of the proposed CNN-BN model with the published results in the literature on the same DEAP dataset. Note that the methods—such as the EEG preprocessing steps and model evaluation—used in previous studies may differ from our methods used in the present study. Nevertheless, a common metric (i.e., accuracy) for performance evaluation can be directly compared for a performance comparison with state-of-the-art models. 

Table 4 presents the performance of our model compared with those obtained by several state-of-the-art models. For the binary classification tasks, the proposed CNN-BN model achieved the state-of-the-art performance on the DEAP dataset. Deep learning models that only consider the temporal or spectral information of EEG signals performed relatively worse, reaching 85.45% for valence and 85.65% for arousal in classification accuracy [15]. Overall, existing emotion recognition methods based on a combination of two or more EEG features have achieved a high classification accuracy (62.27–97.97% for valence and 65.08–98.31% for arousal) [17,19,20,21,22,23,24,25,26,27]. The CNN-BN model achieved superior performance with an accuracy of 99.10% and 99.48% for valence and arousal, respectively, compared to other baselines. This demonstrates the advantages of introducing the channel bottleneck module to the CNN architecture with respect to learning the spatiotemporal EEG features needed for accurate emotion recognition. 

## 6. Conclusions

In this paper, we proposed the 3D CNN-BN model for EEG-based emotion recognition. We introduced the channel bottleneck module to the CNN architecture to reduce the model size and to extract spatiotemporal EEG features, which effectively utilized the spatial and temporal information in EEG. Specifically, the bottleneck blocks were designed to significantly decrease the number of parameters in the model that were required for learning and consequently reduce the computational complexity without losing classification accuracy. The experiments on the DEAP dataset demonstrated that the CNN-BN model with a better parameter efficiency achieved a better performance than state-of-the-art baselines. The CNN-BN model achieved a classification accuracy of 99.10 ± 0.10% and 99.48 ± 0.07% in the valence and arousal classification tasks, respectively. Further, our model with channel bottlenecks reduced the number of parameters by 93.08% and the FLOPs by 94.94% compared to the baseline CNN model. This demonstrates that the proposed model retains classification accuracy, while significantly reducing the computational costs for the acceleration of EEG-based emotion recognition. The CNN-BN model is a general framework based on multi-channel physiological signals, which can be further applied to other applications in the future such as the brain–computer interface, virtual reality, and sleep stage classification. 

## Figures and Tables

**Table 1:** Architectural details of the proposed CNN-BN model for EEG-based emotion recognition. Each row denotes a sequence of building blocks. The proposed CNN-BN model consists of a convolution block, five consecutive bottleneck blocks, and a dense block. The bottleneck building block is repeated five times, denoted as bottleneck block 1 to bottleneck block 5. Each convolutional layer is followed by a batch normalization layer, and the kernel size for convolutions is set to 7 × 3 × 3 or 1 × 1 × 1. The stride of all convolution blocks is set to 1. C, D, H, and W represent the number of channels, length, height, and width of the feature map, respectively. The effects of bottleneck blocks on the number of channels in the output data are highlighted in bold.

Type	Input Size (C × D × H × W)	Channel	Kernel Size	Output Size (C × D × H × W)
Convolution block	1 × 128 × 64 × 64	64	7 × 3 × 3	64×128×64×64
	64 × 128 × 64 × 64	2 × 1 × 1 max pooling, stride 2 × 1 × 1		**64** × 64 × 64 × 64
Bottleneck block 1	64 × 64 × 64 × 64	16	1 × 1 × 1	**16** × 64 × 64 × 64
	64 × 64 × 64 × 64	16	7 × 3 × 3	**16** × 64 × 64 × 64
	64 × 64 × 64 × 64	128	1 × 1 × 1	**128** × 64 × 64 × 64
	128 × 64 × 64 × 64	2 × 2 × 2 max pooling, stride 2 × 2 × 2		**128** × 32 × 32 × 32
Bottleneck block 2	128 × 32 × 32 × 32	32	1×1×1	**32** × 32 × 32 × 32
	32 × 32 × 32 × 32	32	7×3×3	**32** × 32 × 32 × 32
	32 × 32 × 32 × 32	256	1×1×1	**256** × 32 × 32 × 32
	256 × 32 × 32 × 32	2 × 2 × 2 max pooling, stride 2 × 2 × 2		**256** × 16 × 16 × 16
Bottleneck block 3	256 × 16 × 16 × 16	64	1 × 1 × 1	**64** × 16 × 16 × 16
	64 × 16 × 16 × 16	64	7 × 3 × 3	**64** × 16 × 16 × 16
	64 × 16 × 16 × 16	256	1 × 1 × 1	**256** × 16 × 16 × 16
	256 × 16 × 16 × 16	2 × 2 × 2 max pooling, stride 2 × 2 × 2		**256** × 8 × 8 × 8
Bottleneck block 4	256 × 8 × 8 × 8	64	1 × 1 × 1	**64** × 8 × 8 × 8
	64 × 8 × 8 × 8	64	7 × 3 × 3	**64** × 8 × 8 × 8
	64 × 8 × 8 × 8	256	1 × 1 × 1	**256** × 8 × 8 × 8
	256 × 8 × 8 × 8	2 × 2 × 2 max pooling, stride 2 × 2 × 2		**256** × 4 × 4 × 4
Bottleneck block 5	256 × 4 × 4 × 4	64	1 × 1 × 1	**64** × 4 × 4 × 4
	64 × 4 × 4 × 4	64	7 × 3 × 3	**64** × 4 × 4 × 4
	64 × 4 × 4 × 4	256	1 × 1 × 1	**256** × 4 × 4 × 4
	256 × 4 × 4 × 4	2 × 2 × 2 max pooling, stride 2 × 2 × 2		256 × 2 × 2 × 2
Dense block	2048	128D fully connected		128
	128	2D fully connected		2

**Table 2:** Classification performance of the baseline models (LSTM and C3D) and the proposed CNN-BN model for valence and arousal. All performance evaluation results reported are the average (standard deviation) over five individual trials for each model.

Model	Valence				Arousal			
	Recall	Precision	F1-Score	Accuracy	Recall	Precision	F1-Score	Accuracy
LSTM	0.6600 (0.0865)	0.6641 (0.0281)	0.6579 (0.0309)	0.6651 (0.0052)	0.6459 (0.0690)	0.6491 (0.0678)	0.6425 (0.0524)	0.6508 (0.0529)
C3D	0.9888 (0.0004)	0.9888 (0.0004)	0.9888 (0.0003)	0.9890 (0.0003)	0.9925 (0.0011)	0.9930 (0.0022)	0.9928 (0.0024)	0.9929 (0.0023)
CNN-BN	0.9908 (0.0019)	0.9910 (0.0018)	0.9909 (0.0010)	0.9910 (0.0010)	0.9948 (0.0011)	0.9947 (0.0011)	0.9947 (0.0007)	0.9948 (0.0007)

**Table 3:** Comparison of the number of parameters and the number of floating-point operations (FLOPs) between the regular CNN (C3D) model and the proposed CNN-BN model.

Model	Parameters (M)	FLOPs (G)
C3D	16.05	449.29
CNN-BN	1.11	22.74

**Table 4:** Performance comparison of the state-of-the-art models in the binary classification of valence and arousal on the DEAP dataset.

Authors	Feature	Classifier	Accuracy	
			Valence	Arousal
Alhagry et al. [15]	Time-domain signal	LSTM	0.8545	0.8565
Yin et al. [17]	Power, time-domain features	Multiple-fusion-layer based Ensemble classifier of Stacked AutoEncoder (MESAE)	0.7617	0.7719
Sharma et al. [19]	Third-order cumulants (ToC)	LSTM	0.8416	0.8521
An et al. [20]	Bandwise DE 2D representation	CNN-SAE	0.8949	0.9076
Islam et al. [21]	Pearson’s Correlation Coefficient	CNN	0.7822	0.7492
Liu et al. [22]	Time-domain signal	Multi-Level Feature (MLF)-CapsNet	0.9797	0.9831
Sartipi et al. [23]	Graph Fourier Transform Spatiotemporal Attention Neural Network (GFT-STANN)	Spatiotemporal attention neural network (STANN)	0.948	0.961
Yin et al. [24]	DE graph	GCNN + LSTM	0.9045	0.9060
Ding et al. [25]	Time-domain signal	Temporal Spatial Inception (TSception)	0.6227	0.6375
Chao et al. [26]	Multiband Feature Matrix	CapsNet	0.6673	0.6828
Jia et al. [27]	Heterogeneous graph sequence	Graph Transformer Network (GTN), Graph Convolutional Network (GCN)	0.9766	0.9730
Ours	Time-domain signal	LSTM	0.6651	0.6508
	Spatiotemporal 3D representation	C3D	0.9890	0.9929
	Spatiotemporal 3D representation	CNN-BN	0.9910	0.9948

## Data Availability

The DEAP dataset analyzed in this study is available to all researchers and can be assessed upon approval. This data can be found at http://www.eecs.qmul.ac.uk/mmv/datasets/deap/index.html (accessed on 5 September 2022).
